# Design of a lightweight passive orthosis for tremor suppression

**DOI:** 10.1186/s12984-020-00673-7

**Published:** 2020-04-09

**Authors:** Nicolas Philip Fromme, Martin Camenzind, Robert Riener, René M. Rossi

**Affiliations:** 1 Laboratory for Biomimetic Membranes and Textiles, Empa, Swiss Federal Laboratories for Materials Science and Technology, Lerchenfeldstrasse 5, 9014 St. Gallen, Switzerland; 2 Sensory-Motor Systems Lab, Department of Health Sciences and Technology, Institute of Robotics and Intelligent Systems, ETH Zurich, Tannenstrasse 1, TAN E 5, 8092 Zurich, Switzerland; 3 Spinal Cord Injury Center, University Hosptial Balgrist, Medical Faculty, University of Zurich, Lengghalde 5, 8008 Zurich, Switzerland

**Keywords:** Tremor, Suppression, Wearable, Upper limb, Orthosis, Comfort, Textile integrated, Laser welding, Soft, Variable stiffness

## Abstract

**Background:**

Tremor is the most common movement disorder with the highest prevalence in the upper limbs. The mechanical suppression of involuntary movements is an alternative and additional treatment to medication or surgery. Here we present a new, soft, lightweight, task asjustable and passive orthosis for tremor suppression.

**Methods:**

A new concept of a manual, textile-based, passive orthosis was designed with an integrated, task adjustable, air-filled structure, which can easily be inflated or deflated on-demand for a certain daily activity. The air-filled structure is placed on the dorsal side of the wrist and gets bent and compressed by movements when inflated. In a constant volume air-filled structure, air pressure increases while it is inflating, creating a counterforce to the compression caused by bending. We characterised the air-filled structure stiffness by measuring the reaction torque as a function of the angle of deflection on a test bench. Furthermore, we evaluated the efficacy of the developed passive soft orthosis by analysing the suppression of involuntary movements in the wrist of a tremor-affected patient during different activities of daily living (i.e. by calculating the power spectral densities of acceleration).

**Results:**

By putting special emphasis on the comfort and wearability of the orthosis, we achieved a lightweight design (33 g). The measurements of the angular deflection and resulting reaction torques show non-linear, hysteretic, behaviour, as well as linear behaviour with a coefficient of determination (R^2^) between 0.95 and 0.99. Furthermore, we demonstrated that the soft orthosis significantly reduces tremor power for daily living activities, such as drinking from a cup, pouring water and drawing a spiral, by 74 to 82% (*p* = 0.03); confirmed by subjective tremor-reducing perception by the patient.

**Conclusion:**

The orthosis we developed is a lightweight and unobtrusive assistive technology, which suppresses involuntary movements and shows high wearability properties, with the potential to be comfortable. This air-structure technology could also be applied to other movement disorders, like spasticity, or even be integrated into future exoskeletons and exosuits for the implementation of variable stiffness in the systems.

## Introduction

### Tremor

Tremor is defined as the rhythmic and involuntary oscillatory movement of a body part [1]. It is the most common movement disorder in adults and may be a symptom of a disease or the consequence of drugs [1, 2]. For this movement disorder, Essential tremor (ET) and Parkinson’s Disease (PD) are the most common disorder and disease, respectively, and the hands are the most affected site [3–6]. Tremor can be distinguished between rest and action tremor; action tremor can be further classified into different sub groups, e.g. postural, kinetic and isometric tremor [7]. ET is the most prevalent type, with 4.6% of the population aged 65 and older affected, causing tremor in the upper limb. PD develops in 2% of people older than 65 [8, 9]. In the overall population of the USA, approximately 2.2% (7 million individuals) are affected by ET [10]. More than 65% of those suffering from tremor in the upper limb present serious difficulties in performing activities in daily life (ADL) [6, 11]. Furthermore, 34% of ET and 48% of PD patients were found to be at least mildly depressed [12].

### Problem definition

Currently, neither ET nor PD are curable and, therefore, treatment is focused on relieving the symptoms to increase the quality of life of the patients [13]. Even though up to 53% of patients discontinue medication because of side effects or lack of efficacy, it is the most commonly used treatment for tremor [14, 15]. In severe cases, a surgical and invasive intervention (such as Deep Brain Stimulation) can be performed [3]. Further surgical treatments and interventions that are being researched include radiofrequency lesioning, gamma-knife radiosurgery and high intensity, focused ultrasound [16–18].

As a large fraction of patients are either refractory to medication (50% of ET patients), not qualified for surgical treatment or drug intolerant, alternative treatments are needed. Such alternative and supplementary treatments include functional electrical stimulation, sensory electrical stimulation, limb cooling and vibration therapy. Intervention with an external force, i.e. tremor suppressing orthoses, is a straightforward treatment with the potential for high tremor suppression efficacy (for the suppression of tremor in ADL, particularly action tremor).

### State of the art

Several publications have presented different approaches for tremor suppressing orthoses. These orthoses can be classified by the type of tremor suppression mechanism employed: passive = energy dissipation and/or absorption, semi-active = active controller adjusted energy dissipation and/or absorption, and active = force inducing. The most prevalent approach is an active orthosis [19]. However, none of the presented systems reached the market, because the solutions presented showed drawbacks, such as too much weight and restrictions in degrees of freedom (leading to patient rejection in some cases) [20]. Low patient acceptance can be explained by bad wearability [19]. The majority of existing suppressing orthoses are bulky and have rigid structures, to which a wearer is burdened with an additional weight of 20% on the arm, not considering the energy source and control unit of the active and semi-active orthoses, leading to muscle fatigue [21]. Furthermore, the patient’s unknown voluntary motion intention complicates control in active orthoses, compared to traditional stabilisation or tracking control. It can, therefore, be hypothesised that a tremor suppression orthosis with better wearability and lower weight will be better accepted by the wearers, while having a similar tremor absorption efficacy of 67% [19]. Passive and even semi-active mechanisms are often lighter and less cumbersome compared to force-inducing mechanisms. Classical passive devices usually rely on energy dissipation mechanisms, such as:

- an air dashpot orthosis, relying on an attached piston coil system [22],
- an orthosis with a rotary damper [23],

and/or energy absorption mechanisms, such as:

- the “Vib-Bracelet”, a tuned spring-mass damper [23, 24].

Pneumatic mechanisms are the most prevalent system in hand rehabilitation and assistance but most of them are force-inducing [25]. Mass, damping, and stiffness represent the combined properties of the human limb system, while increasing joint stiffness reduces undesirable involuntary movements [26]. However, changes in mass and damping properties also affects tremor intensity.

Kalaiarasi et al. developed a variable-stiffness air cuff system relying on inflatable air handcuffs using an accelerometer-threshold based control [27]. They can achieve 31% tremor acceleration amplitude reduction, whilst maintaining that voluntary movements in the arm are unaffected without providing any validation of the voluntary movements [27]. However, the used handcuff is not delineated, nor is the material used, its weight, geometry, positioning or appearance. Based on the picture of the setup, the used cuff was most probably a commercially available inflatable cast or similar. Thus, their system is clearly different from the one described in this study, as we present a passive, soft, textile integrated system with special emphasis on wearability and low weight for ADL. Furthermore, the user can autonomously enable the orthosis by inflating the air-filled structure for a certain task or situation.

### Objective

A new design for an orthosis needs to combine a user’s comfort, as well as wearability, in order to achieve a high acceptance in ADL. Comfort is a subjective state, based on physical, physiological and psychological factors [28]. The wearability of the device combines different criteria, including performance efficacy, usability, appearance and reliability to reach a sufficient acceptance by the user.

#### Comfort

In general, comfort is defined as an absence of discomfort and pain [29]. Comfort consists of four different types: psychological comfort, thermophysiological comfort, sensory comfort and ergonomic comfort [30]. Thermophysiological comfort is the ability of the body to balance the production and loss of body heat [30]. Here, a composition of skin-friendly textiles with a Resistance to Evaporating Heat Transfer (RET) after ISO 11092 of less than 21 m^2^Pa/W, showing high comfort in medical applications, is desired [31–33]. Sensory comfort includes tactile, thermal and moisture sensation, whereas for ergonomic comfort clothing has to incorporate mobility features [30]. For tactile sensation, mechanical parameters such as shear loads, friction and normal loads are decisive, whereas shear loads seem to have the highest impact on comfort [34]. Ergonomic comfort, or biomechanical comfort, includes fitting to the human shape and not restricting voluntary movements, degrees of freedom, or the natural workspace while still being lightweight. An individual adaptation of the device to a user’s anthropometry ensures an optimised fit. The mass should be as lightweight as possible and the size as small as possible [19]. Apart from a high level of comfort, a lightweight design will also prevent muscle fatigue, which is of special importance for tremor patients because of their advanced age and the associated degenerative loss of skeletal muscle mass [19].

#### Wearability

Besides the device’s performance efficacy, three other human-centred design principles need to be considered for a functional orthosis with high acceptance. The usability of the device includes the intuitiveness, ease of use (like donning/doffing) and interaction [35]. Reliability conflates the confidence and trust in the device by the user which primarily combines the safety and robust tremor suppression efficacy of the device. Here, aesthetics not only include the visual appeal of the orthosis but also the individual adaptation of the device features (such as inflation system or individual design) to the user’s preferences and wishes.

In this paper, we describe the development of a mechanism for a manual Task-Adjustable Passive Orthosis (TAPO). This mechanism is based on an air-filled structure, relying on a working principle similar to Tensairity® [36]. It is a bionic mechanism, which stabilises by pressure, in a similar manner to plants which are stabilised by turgor–pressurised structures [36] or worms, starfish feet and sharks [37]. The concept of task-adjustable variable stiffness is inspired by the octopus, which also changes its tentacle segment stiffness depending on its needs. To the best of our knowledge, no such task-adjustable passive device has been described in previous literature.

## Methods

### System design

The proposed orthosis was designed as a textile glove with a specific focus on high wearability (i.e. skin-friendly textiles, low weight and easy donning/doffing) and user-adjustability for specific tasks. The variable stiffness is obtained through an air-filled membrane structure which can be inflated or deflated by the user, on-demand. Thus, the air-filled structure is only inflated when tremor suppression is needed. Our concept foresees two inflating mechanisms, either by hand or electrical pump.

**Fig. 1:**
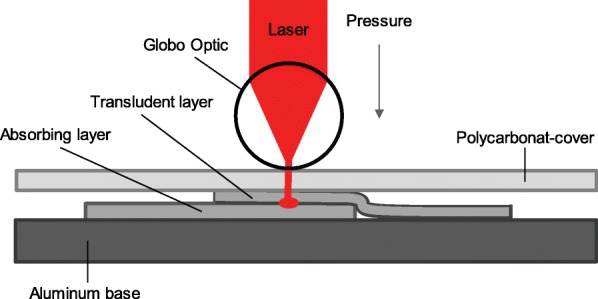
Laser welding setup of two layers

For the development of the air-filled structure, a newly-developed laser welding technique was used [38]. With this technique we can bond two polymeric membranes or textiles using an infrared laser. For this process it is important to have an infrared-absorbing layer, which converts the energy into local heat, as well as a translucent layer, which allows the laser to be transmitted to the absorbing layer [39]. The heated material will partially melt to get the bonding. The two layers need to originate from the same polymer family for layer fusion, in addition to being mechanically stable in order to achieve a flexible welding seam. Two partially absorbing and translucent membranes of 1 mm thickness (Sympatex Technologies GmbH, Unterföhring, Germany) were chosen for their mechanical robustness, and good thermal and moisture management. The laser (of wavelength 940 nm (NOVOLAS Basic AT Compact, Leister Technologies AG, Kaegiswil, Switzerland)) is directed to the materials by light guides and focused with a Globo Optic (Leister Technologies AG, Kaegiswil, Switzerland). The Globo Optic has an air-suspensioned sphere, applying a pressure of 1 bar on the two membrane layers. This optical system, mounted on a XY CNC table (Gunnar AG, Altstätten, Switzerland), is controlled by a PC with Wingman 2.5 software (IsoDev GmbH, Wegscheid, Germany). On top of the two membrane layers, we used a transparent polycarbonate cover in order to keep the layers in place and for a constantly smooth surface structure for the Globo Optic (see Fig. 1). A laser power of 7 W, at a spot size of 3.14 mm^2^ with a forward feed of 10 mm/s, was identified as the functional setting by preliminary tests.

**Fig. 2:**
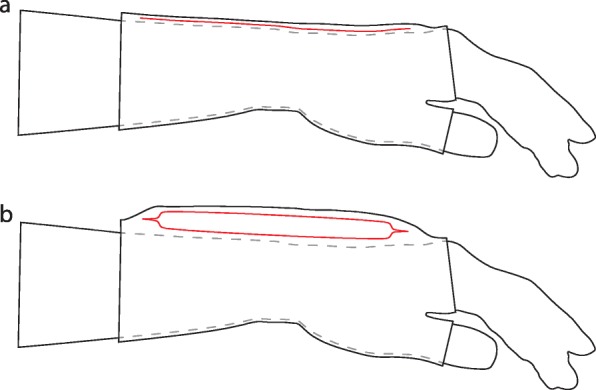
Location of the air-structure (red) in deflated (**a**) and inflated (**b**) state at the dorsal side of the wrist with the glove for positioning

**Fig. 3:**
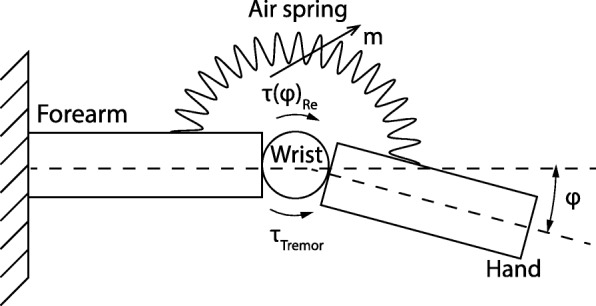
Mechanical working principle model of the air-structure based wrist tremor suppression system with hand connected to the forearm by the wrist. The reaction torque τ(φ)_Re_, as a function of the air springs mass (m), is a response to the deflection resulting from the overall wrist dynamics, including human joint and orthosis dynamics and the tremorous torque τ_Tremor_

This versatile laser welding technique enabled us to develop air-filled structures with different designs and materials, creating any 3D shape which can be implemented into conventional clothing. However, similar, more elaborate and less versatile welding results could be obtained by a conventional method such as a hot press. Tremor in the upper limb is concentrated in the wrist flexion-extension φ, ulnar-radial deviation ϑ and forearm pronation-supination, while other DOF are not negligible [40]. For proof of the concept, we decided to manufacture a simple cylindrical shape; the structure was especially designed for placement on the dorsal side of the wrist and integrated into a textile glove (see Fig. 2). The air-structure acts as a tuneable (air mass dependent) air spring, creating a reaction torque τ(φ)_Re_ in response to the deflection resulting from the overall wrist dynamics, including the stiffness of the orthosis, human joint dynamics and the tremorous torque τ_Tremor_ (see Fig. 3).

For our demonstrator, a unified air-structure is designed to fit all. Therefore, the 10 percentile values of the hand’s anthropometry (by Wagner) are chosen as wrist dimensions [41]. In order to reduce normal loads on the soft tissue, a large surface is desired on which the air-structure distributes its force on. The average 10 percentile length of the back of the hand is 83 mm [41], so we decided to cover not more than 90% (75 mm) of it, so as not to interfere with the metacarpophalangeal (knuckle) joint. For a symmetric pressure distribution at the back of the hand and the forearm, a length of 150 mm for the air-structure is chosen (i.e. twice the 75 mm distance). The wrist breadth of 51 mm [41] and the design choice to cover not more than 75% of it, led to a width of the air-structure of 35 mm. This design choice is based on the shape of the lower forearm, where the curvature increases towards the radial and ulnar side of the forearm. On the other hand, it maximises the force induced surface, while minimising the covered skin by stacked layers of membranes and textiles, for thermal reasons.

Sharp edges and small radii had to be avoided during the laser processing, to prevent the local heating from becoming too high and destruction of the membrane, due to a decrease of the laser forward feed. Furthermore, the rounding of corners with too small radii can cause the corner to protrude when inflated. This optimisation work led to air-filled structures with inner dimensions of 150 mm (length) and 35 mm (width) with 10 mm rounded corners, obtaining a cylindrical shape of 24 mm diameter and 150 mm length, when inflated.

**Fig. 4:**
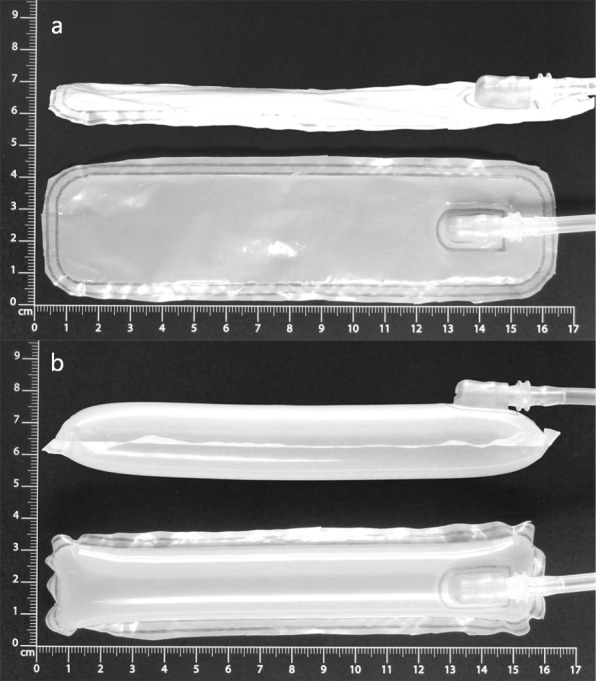
Top and side view of the air-structure while inflated and deflated. **a**: deflated air-structure. **b**: inflated air-structure

**Fig. 5:**
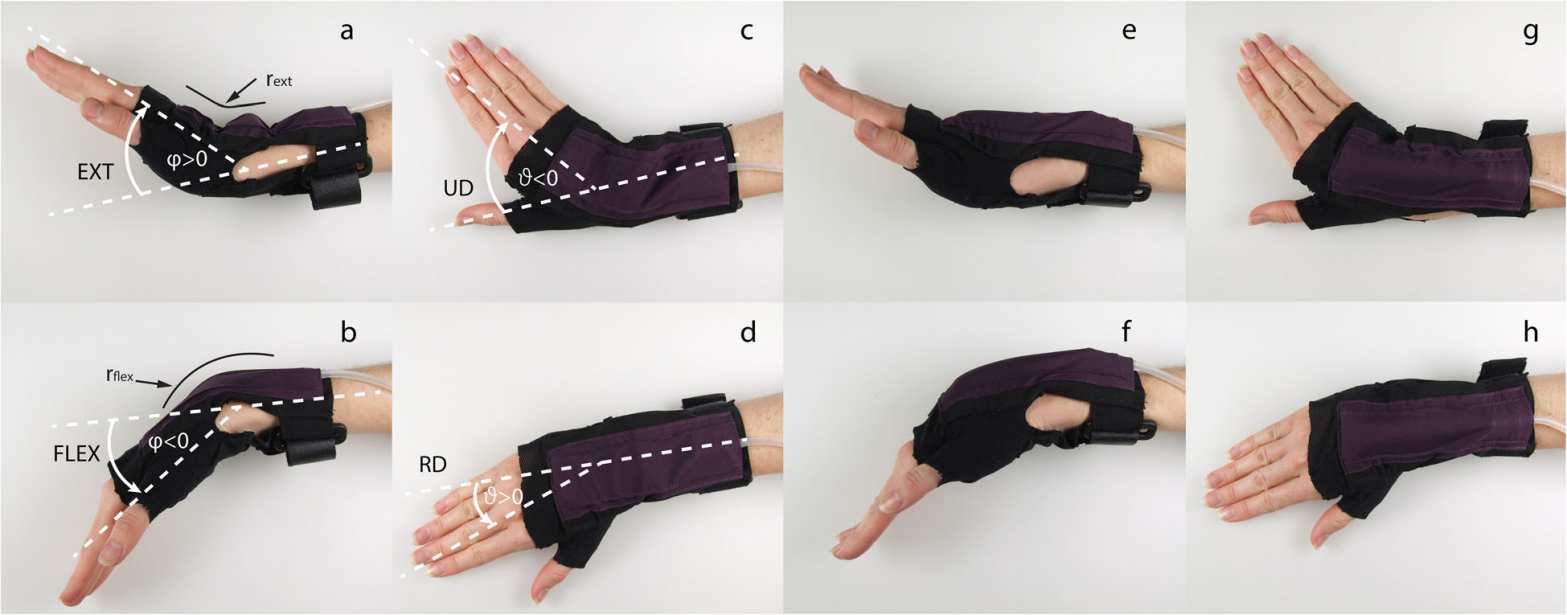
Deflated orthosis in **a, b, c, d** and inflated orthosis in **e, f, g, h** in extension, flexion, ulnar and radial deviation, respectively. **a, b, c, d** indicate the angles φ > 0 for extension (EXT), φ < 0 for flexion (FLEX), ϑ for ulnar deviation(UD) and radial deviation (RD). The bending radius of the air-structure given by the wrist dimensions is illustrated in **a** and **b** with r_ext_ and r_flex_

The designed orthosis mechanism relies on an air-structure air-spring, as shown in Fig. 4. The glove is fixed to the hand (free fingers) with a slightly flexible tight fit; after a wide opening at the forearm (for an easy don), velcro fasteners were used for a tight fit (see Fig. 5). The don and doff movement is simplified, compared to a conventional glove because one big outlet for all of the fingers was integrated into the glove. The materials used in the glove are polyamide at the hand palm (because of its robustness) and polyurethane and spandex at the back of the hand (for its elasticity and thermal properties). This has a polyamide extension attached to the forearm. In the extension, a pocket was integrated for the air-structure, made out of a breathable polyester membrane. The total weight of the orthosis with air-structure is 33 g.

### System properties

**Fig. 6:**
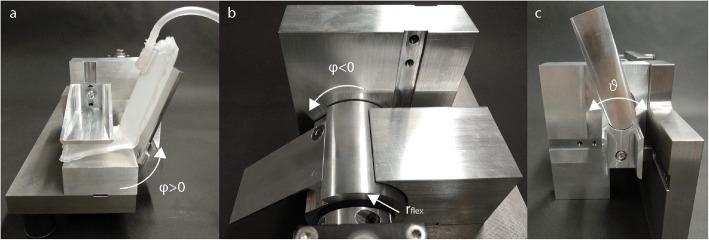
Air-structure test bench for extension φ > 0 in **a**, flexion φ < 0 in **b** and ulnar-radial deviation ϑ in **c**. The Figure shows a single device from different angles, whereas c is a different configuration of a. The air-structure in a shows a fold occurring when deflected φ > 30°. In figure **b** the estimated wrist flexion radius r_flex_ of 22 mm can be seen

We characterised the air-filled structure to suppress tremor by defining the relation between angular deflection and reaction force and measuring the resulting stiffness of the air-structure. Therefore, we assumed that a simplified model of the wrist joint can be created by hinges. For this purpose, a test bench was designed (see Fig. 6) with an estimated outer flexion radius r_flex_ of 22 mm (see Fig. 5b) which corresponds to the flexion on the dorsal side of the wrist and defines the bending of the air-structure during flexion (based on an internal study with 15 healthy subjects). The radius r_ext_ arising during extension at the wrist (see Fig. 5a) was neglected because it is approximately zero and the air-structure bending radius is not defined by the wrist. The air-structure was guided on one side of the test bench, the other side being fixed with velcro. With an external force introduced by a wrench, we continuously deflected the air-filled structure, while measuring the angle and the torque simultaneously, using a precision torque sensor (8661–5005-V0400 with angle measurement, Burster Präzisionsmesstehnik GmbH & Co. KG, Germany). The measurement procedure was repeated for flexions from − 90° to 0°, extension from 0° to 90°, ulnar deviation from − 50° to 0° and radial deviation from 0° to 50°; this was carried out five times (back and forth) as well as for different inflation states. The different states were: X: deflated (0 ml air), A: 1.1 bar (40 ml air), B: 1.35 bar (60 ml air) for absolute pressure as well as a fully inflated state C (with a maximum absolute pressure of 1.5 bar (80 ml air), adjusted by manual inflation with a syringe). The measured deflection angles cover the range of motion for ADL of − 40° to 38° (for wrist flexion-extension φ) and − 28° to 38° (for wrist ulnar-radial deviation) [42]. Natural wrist movements include many small angular displacements of the wrist. Therefore, we analysed the characteristics of the air-structure for angular displacements of ±6° of a 25° position for flexion, extension and ulnar-radial deviation. The measurement was repeated for a frequency of 1 Hz, for natural movements, and 5 Hz, for involuntary movements, for 20 s to differentiate the characteristics for the different movement types and dynamics, respectively. For the stiffness measurements, velocity was kept below 1 deg/s in order to minimise dynamic influences, like mass inertia, for the characterisation. The measured data were processed and analysed with Matlab (The MathWorks Inc., Natick, MA, USA). From the measured angle-reaction torque relation, a fitting linear model was derived for the motion ranges. This was to calculate the stiffness of the air-structure by assuming a linear relationship for ulnar-radial deviation ϑ and flexion-extension φ of the air-structure stiffness in the range of motion for ADL, due to the linear air pressure-volume relationship for small compressions. The hysteresis for the small angular displacements was first analysed by creating a 4th grad polynomial model of the curve and then determining the maximum stored energy (W) as well as the energy losses (ΔW) by calculating the hysteresis area. The damping ratio (D) of the stored energy and ratio of energy loss were then calculated (see Eq. 1).

1 $$ D=\frac{1}{4\pi}\frac{\varDelta W}{W} $$

### Proof-of-concept

In order to analyse the TAPO demonstrator with the laser welded air-structure under realistic conditions, its performance was examined with regard to one patient during selected ADL, based on standardised motor tasks from the WHIGET Tremor Rating Scale Test [43]. WHIGET is a clinically established and recommended rating scale for tremor consisting of the following tasks: drawing a spiral (Drawing-Spiral), pouring from a bottle into a glass (Pouring), drinking from a glass (Drinking), using a spoon (Spoon-Up), arm extension (Arm-Extension) and finger-to-nose movement (Finger-to-Nose). Each task in a phase is repeated 4 times in a row to compensate for singularities in a movement and to receive an average movement sequence. One phase with the movement sequence tasks was performed with a deflated (disabled) glove while the other phase had the same sequence order but with an inflated (enable) glove. The tests all took place during the same day. The movement was tracked with an electromagnetic motion tracking system (LIBERTY, Polhemus, USA), whereas the tremor was rated simultaneously with the WHIGET scale. With the orientation of the hand and forearm in relation to the base (Euler angles), the wrist joint angles can be calculated. Matlab was used for signal post-processing to determine the angular accelerations for the calculated wrist joint position angles and the given sample rate of 240 Hz. The signal was filtered with a high-pass Butterworth filter at 0.1 Hz and a low-pass Butterworth filter at 15 Hz, to suppress movement artefacts. Furthermore, the power spectral density (PSD) for the wrist radial and ulnar deviation, wrist flexion and extension, pronation and supination was calculated, as well as the absolute hand movement (relative to the electromagnetic motion tracking base and table, respectively). This determines the tremor power, defined as the power of the angular acceleration [deg^2^/s^3^] over its frequency in Hertz. The PSD was calculated using the Welch-Bartlett Method (based on fast Fourier transform) using a 50% overlap of 500 sample Hamming windows [44], which is the most common method for tremor analysis [45]. To determine the power reduction of tremor and voluntary movements, the dominant tremor power frequency was identified in the range of 3 to 12 Hz [4], whereas the power peak of natural movements was identified in the range of 0 to 2 Hz [46] for inflated and deflated orthosis.

For PSD referencing, the performance of the orthosis was further evaluated by analysing the drawn spirals as an objective, visual representation of tremor intensity [47, 48]. The spirals were evaluated using a scan of the drawings to analyse the length of the spiral with inflated and deflated orthosis, using a vector graphics editor (Adobe Illustrator CC 2015.3, Adobe Inc., San Jose, CA, USA). Statistical significance was determined for all measurements using the Mann-Wilcoxon-U-Test with a significance level of *p* ≤ 0.05.

The patient was a 76 year old male participant, who had suffered action and rest tremor for 16 years (caused by PD), although he was on medication. For the study session, the patient continued his medical treatment as usual. Furthermore, he was free of any other conditions affecting upper limb movement or motor control (e.g. spasticity, paralysis or muscular insufficiency) and had no known injuries or illnesses that may have affected safe participation.

## Results

### System properties

**Fig. 7:**
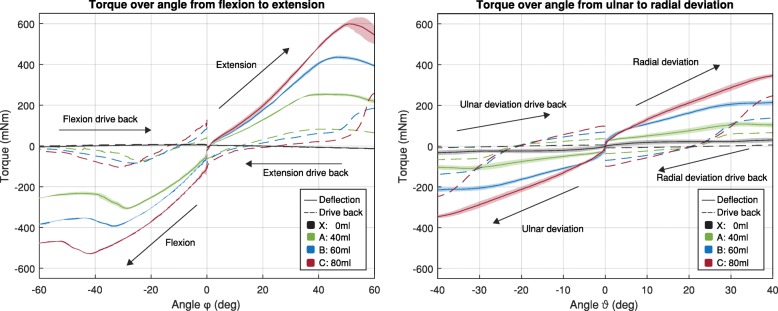
Left: Reaction torque of the air-structure in wrist flexion-extension direction and drive back on the test bench. Right: Reaction torque of the air-structure in wrist ulnar-radial deviation direction and drive back on the test bench. Black line represents the air-structure in deflated state X: 0 ml air, green line represents an air-structure filled with A: 40 ml air (initial pressure of 1.1 bar), blue with B: 60 ml air (initial pressure of 1.35 bar) and red C: 80 ml air (initial pressure of 1.5 bar). The standard deviation of flexion, extension, radial and ulnar deviation is indicated with a shade

The relation between angular deflection and reaction torque of the air-structure shows linear behaviour in flexion-extension φ and ulnar-radial deviation ϑ from 0° to ±30°, whereas flexion and extension (as well as extension drive back) show reduced torque after 30° (see Fig. 7). The torque jump and hysteresis at 0° is related to its damping properties, characterised by the small displacement experiment. The slope of flexion, extension radial and ulnar deviation corresponds to the stiffness in this degree of freedom. The coefficient of determination, R^2^, for the linear model of flexion-extension for all three inflation states (between 0° and ± 30°) ranges from 0.98 to 0.99. The coefficient of determination for the radial and ulnar deviation of all three inflation states between 0° and 30°, ranges from 0.95 to 0.99. During extension and extension drive back, the torque drop starts at 30°, depending on the inflation state, whereas flexion shows a similar drop starting from − 30°, depending on the inflation. This drop in torque was observed to be related to a fold in the air-structure which occurred during bending. The reaction torque was the highest for the most inflated structure (state C: 80 ml) and reached − 530 mNm for the flexion and 600 mNm for extension. Ulnar-radial deviation reached up to 340 mNm for state C. The extension and flexion drive back torque were much lower and did not exceed 0.1 Nm from 0° to ±50°. From − 50° to − 60°, the flexion drive back force is close to 0 Nm, whereas the extension drive back increases to over 200 mNm, between 50° and 60°.

Based on these results and the resulting linear models, the stiffness was estimated for the wrist at the three different inflation stages: A, B, and C. Table 1 compares the estimated stiffness of the air-structure to the passive stiffness of the human wrist from the study carried out by Formica et al. [49] and the 1-DOF stiffness of commercially available, medical, dorsal orthosis Smart Glove, IMAK and The Clutch, PRO-TEC ATHLETICS [50].

**Table 1:** Stiffness of air-structure at the inflation stages A, B, C, compared to human wrist stiffness and two medical dorsal orthosis for wrist deflection in flexion, extension, ulnar and radial deviation and the drive back

Movement	Stiffness in mNm/rad					
	Air-structure			Human wrist [49]	Smart Glove, IMAK^a^ [50]	The Clutch, PRO-TEC ATHLETICS^a^ [50]
	A:40 ml	B:60 ml	C:80 ml			
Flexion (φ < 0)						
Deflection	507	620	667	554	200	1500
Drive back	220	325	408			
Extension (φ > 0)						
Deflection	404	560	633	1021	200	1500
Drive back	230	191	159			
Radial deviation (ϑ > 0)						
Deflection	152	320	493	1710	300	1500
Drive back	152	300	334			
Ulnar deviation (ϑ < 0)						
Deflection	152	320	493	1245	300	1500
Drive back	152	300	334			

^a^ 1-DOF Stiffness converted from 2-DOF stiffness ellipse parameters (orthosis #4 and #6)

**Fig. 8:**
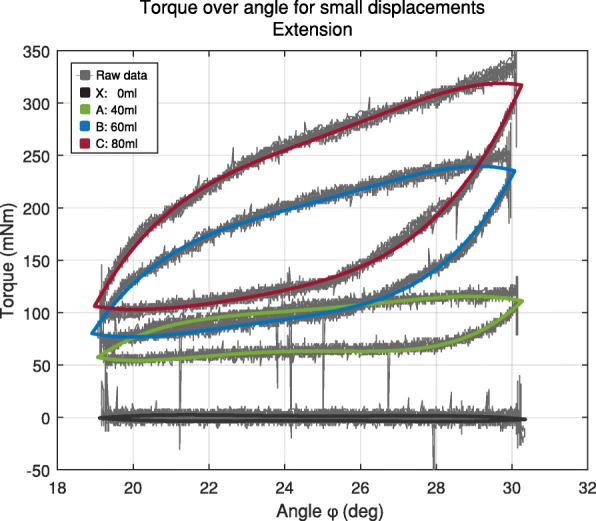
Hysteresis examples of the angular displacement of the air-structure and the corresponding counter torque for the three inflation stages a: 40 ml, b: 60 ml and c: 80 ml at 1 Hz

The displacements for large angles, as well as the displacement for small angles, show a large hysteresis (see Fig. 7 and Fig. 8). The energy storage (W) of ulnar-radial deviation ϑ and extension φ > 0 are within the same magnitude, whereas flexion φ > 0 has high energy storage. Additionally, flexion φ > 0 shows the highest amount of lost energy (see Table 2).

**Table 2:** Air-structure hysteresis properties of small angles of ulnar-radial deviation ϑ, extension φ > 0 and flexion φ < 0. The properties ΔW for the energy loss, W for the stored energy and D as damping ratio are calculated for the inflation state A: 40 ml, B: 60 ml and C: 80 m for with a deflection of ±6° around 25° for 1 Hz and 5 Hz

	Ulnar-radial deviation ϑ			Extension φ > 0			Flexion φ < 0		
	ΔW (mNm rad)	W (mNm rad)	D	ΔW (mNm rad)	W (mNm rad)	D	ΔW (mNm rad)	W (mNm rad)	D
X: 0 ml									
1 Hz	0.64	0	/	0.07	0	/	0.85	0	/
5 Hz	3.22	0	/	1.05	0	/	4.29	0	/
A: 40 ml									
1 Hz	36.08	9.76	0.59	6.25	6.80	0.15	68.83	49.05	0.22
5 Hz	35.88	17.87	0.32	6.96	17.72	0.06	61.42	49.76	0.20
B: 60 ml									
1 Hz	59.86	12.60	0.76	10.62	16.05	0.11	88.25	55.53	0.25
5 Hz	62.38	19.71	0.50	11.48	24.12	0.08	80.06	66.38	0.19
C: 80 ml									
1 Hz	77.38	13.66	0.90	9.51	20.17	0.07	97.91	60.99	0.26
5 Hz	76.94	23.07	0.53	11.62	26.09	0.07	102.39	78.30	0.21

### Proof-of-concept

**Fig. 9:**
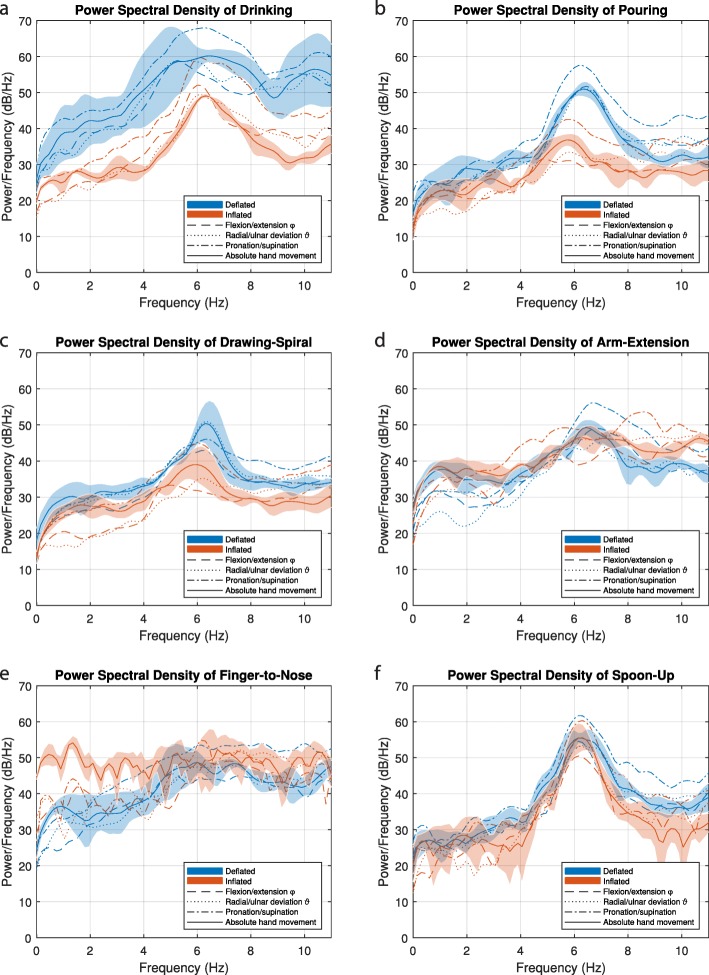
Power Spectral Density of the wrist movement with deflated and inflated orthosis for the tasks **a**-Drinking, **b**-Pouring, **c**-Drawing-Spiral, **d**-Arm-Extension, **e**-Finger-to-Nose and **f**-Spoon-Up plotted for flexion-extension, ulnar-radial deviation, pronation-supination and absolute hand movement with standard deviations as shade

**Fig. 10:**
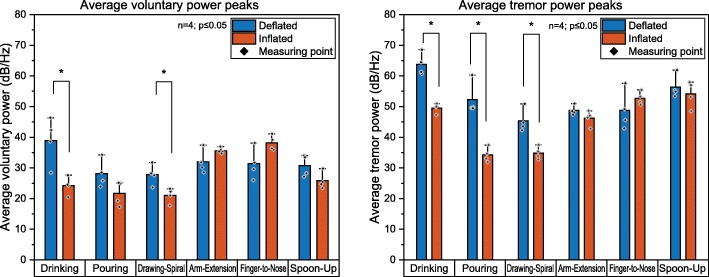
Average power with inflated and deflated orthosis, with significant power reduction in tremor (*p* ≤ 0.05) for Drinking, Pouring and Drawing-Spiral (right) and in voluntary movements for Drinking and Drawing-Spiral (left)

The PSD of the absolute hand movement is compared for each task, between inflated and deflated TAPO as well as for flexion-extension, ulnar-radial deviation and pronation-supination (see Fig. 9). The PSD at the peak frequency from tremor and voluntary movement of the exercise are compared in Fig. 10. For the tasks Pouring, Drawing-Spiral, Arm-Extension, and Spoon-Up (Fig. 9b, c, d and f), the tremor peaks are between 6.0 and 6.5 Hz. Drinking shows the strongest significant decrease of tremor power from 63 dB to 49 dB (82%) and *p* = 0.03. For the Finger-to-Nose task, no significant tremor reduction was found (*p* = 0.34) but a frequency shift from 5.5 Hz to 7.5 Hz was observed (Fig. 9e). The inflated orthosis did not reduce power for the Spoon-Up task (*p* = 0.69). Further significant tremor power reduction for the tasks of Pouring (79% and *p* = 0.03) and Drawing-Spiral (74% and *p* = 0.03) were found. For Drinking, an overall constant power reduction of the entire curve, including voluntary movements, was observed. The tremor peak reduction performance for the Drinking, Pouring and Drawing-Spiral tasks ranges from 74 to 82%, whereas for the other three tasks, no significant tremor reduction was found. Voluntary movements, in the frequency spectra of PSD from zero to 2 Hz, have been significantly reduced by the orthosis for Drinking (69% and *p* = 0.03), whereas the voluntary movement increased significantly for Finger-to-Nose by a factor of 7 in the absolute hand movement but not in the wrist, relative to the forearm. Furthermore, no distal to proximal tremor shift was visually observed in this case study.

**Fig. 11:**
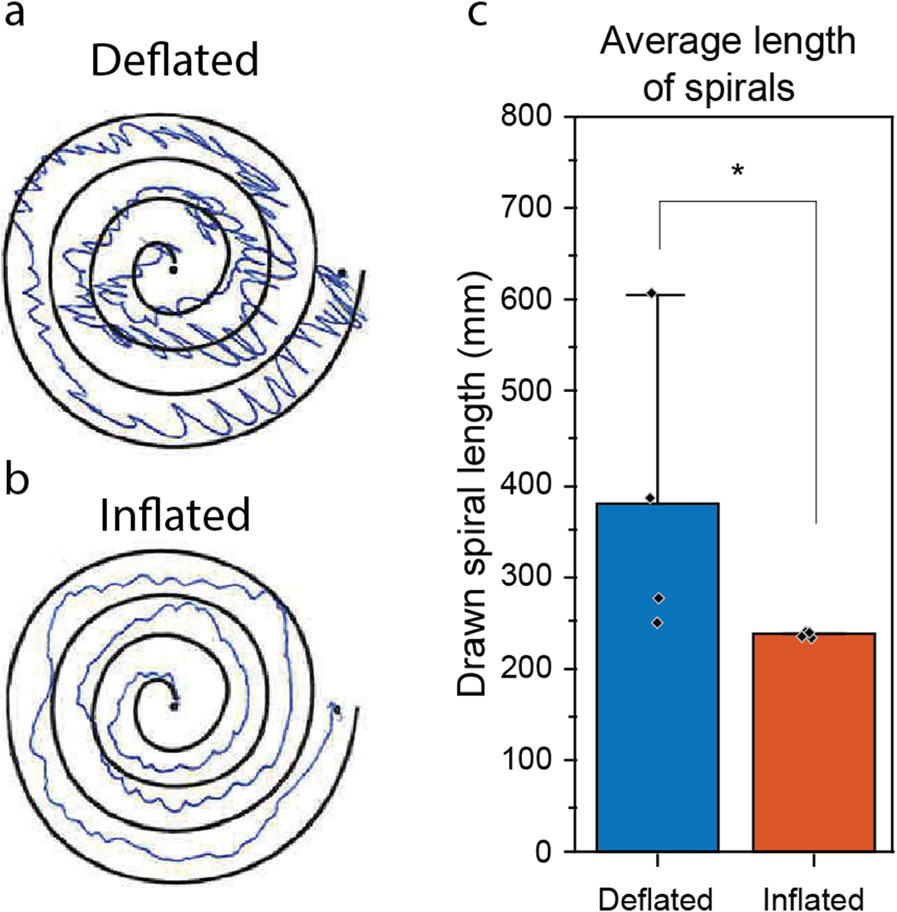
Example of drawn spirals for orthosis inflated and deflated and its average length. **a**: A drawn spiral when orthosis deflated and **b**: when inflated. **c**: The bar diagram of the mean length change of the drawn spirals (measuring points marked by dots)

The length of the spirals drawn with inflated and deflated orthosis has been reduced significantly by 38% (see Fig. 11). With an inflated orthosis, it can be observed that the drawn lines stay between the boundaries of the spiral for most of the time. Additionally, involuntary drawn circles interrupting the drawn line, resulting from high tremor amplitudes, are not observed when the orthosis is inflated.

**Fig. 12:**
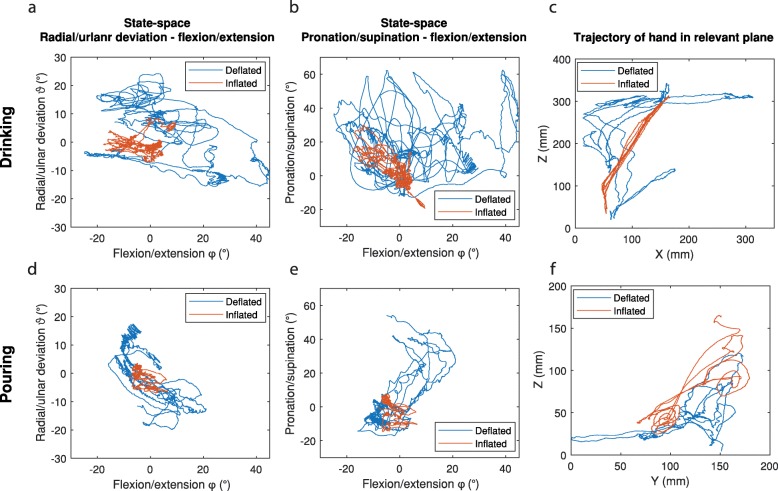
Trajectory of the hand for the Drinking task in **a, b, c** and Pouring in **d, e** and **f**. The angular wrist movement in state-space of ulnar-radial deviation ϑ over flexion-extension φ in **a, d** and pronation-supination over flexion-extension φ in **b, e**. In **c** and **f** the trajectory of the hand in space is plotted in the relevant plane XZ and YZ respectively

For the Pouring and Drinking task, the state-space of ulnar-radial deviation ϑ over flexion-extension φ and pronation-supination over flexion-extension φ, as well as the trajectory of the absolute hand movement in the relevant plane, is shown in Fig. 12. With an inflated orthosis, it can be seen that the range of motion of the wrist is reduced, whereas the trajectory appears more aimed and straightforward, with less detours. The patient claimed to perceive general tremor suppression when wearing TOPAS.

## Discussion

### System properties

The experiments were performed on a test bench, without the interaction and influence of a wearer. A human user would be likely to increase the variability of the data, as every wearer contributes differently to each repetition. Furthermore, every human might influence the system’s behaviour differently because the human limb acts as a parallel, passive mass, and adds spring damper elements to the orthosis.

The characterisation of the newly developed, laser-welded air-structure shows linear behaviour between the reaction torque and the angle from 0° to ±30° for wrist ulnar-radial deviation and extension, over which the slope can be determined by linear regression (R^2^ ≥ 0.95). This relation comes from the linear relation of air pressure-volume for small compressions, as given by the ideal gas law. We hypothesise that the stiffness differences between flexion and flexion drive back, as well as between extension and extension drive back are due to slight displacements of the air-structure and to viscoelastic properties of the membrane. The implementation of a pivot with a distance of 22 mm (to simulate the natural wrist bending radius (rounded corner) on the dorsal side) caused no stiffness difference for flexion to be identified. The analysis of the damping hysteresis shows that the absorbed and stored energy is the highest in flexion. The air-structure attached to the dorsal side of the wrist is further away from the pivot and has a defined bending angle on the surface of the implemented extension circumference, compared to extension. We hypothesise that the low drive back torque in the orthosis leads to a suppression of the tremorous deflection without further inducing force in the upper limb, when driving back from this involuntary movement.

We observed that, when starting from ±30°, the drop in torque during flexion, extension, flexion drive back and extension drive back depended on the inflation state and was due to a created fold in the air-structure. The interactions of the glove with soft human tissue and biomechanics are not included in these measurements. This interaction will influence the resulting stiffness to be added to the biomechanics because the stiffness added to the human skeletal system is caused by the soft tissue, consisting of muscle, skin, fat, ligaments, blood vessels, nerves and tendons which show a combination of non-linear, elastic and viscoelastic elements [51].

The stiffness of the air-structure, when fully inflated, is within the range of commercial, medical, dorsal orthosis. In flexion and extension, the stiffness is higher compared to ulnar and radial deviation, whereas the stiffness of the human wrist is vice versa (see Table 1). We hypothesise that this property adds more stiffness to the human low stiffness flexion and extension compared to the stiff ulnar and radial deviation, leading to a compensation of asymmetry in human wrist stiffness and reducing the deflection amplitude by tremulous muscle activation on the low stiffness side. Tremorous torque direction (and propagation of previous joints) is most likely to be asymmetric, while the deflection and amplitude is the product of the muscle torque and the transfer function of the joint dynamics. Depending on the muscle torque direction, its caused deflection direction is shifted towards the nearby DOF with the lower resistance and stiffness, respectively.

The air-structure shows linear stiffness properties as well as non-linear damping properties.

The damping energy loss at 1 Hz compared to 5 Hz shows high similarities. It is observed that the stored energy at 5 Hz is higher, which can only be explained by the dynamic behaviour of the accelerated test bench mass (kinetic energy). The similarity in damping energy loss leads to the conclusion that the air-structures damping is hysteretic in nature, meaning that the energy dissipation is dependent on the lead level and independent from the frequency. Compared to the hysteretic damping property the viscous and coulomb (friction) damping are negligible. This property can be lead back to the heat energy loss of the mem-brane and the compressed air as well as the damping properties of the membrane-structure.

Furthermore, flexion φ < 0 has energy losses, and stores energy of a higher magnitude, compared to the ulnar-radial deviation and flexion. This could be due to the fact that, for flexion, the air-structure is loaded differently and this is caused by the increased wrist bending radius r_flex_.

### Proof-of-concept

This proof-of-concept on one patient showed that TAPO reached a tremor suppression of up to 82% (for the Drinking task) from the tremor peak with deflated orthosis to inflated orthosis. TAPO’s tremor suppression performance on the patient varied between the 6 tested ADL. No significant suppression of the peak tremor power was measured in the Spoon-Up task, where the wrist flexion (and supination) is the highest of all six tasks [52]. We hypothesise that the high supination causes a volume change at the forearm, leading to a reduced transition of forces, while at the same time the high flexion angle forces the air-structure to fold when the resistance force is reduced (see Fig. 6). The tremor reduction in the Drawing-Spiral task is confirmed by the significant reduction of the length of the drawn spiral. The increased voluntary movement in the Finger-to-Nose task could be caused by an increase of movement from other joints of the arm, since voluntary movement did not increase for flexion-extension φ, ulnar-radial deviation ϑ nor pronation-supination (see Fig. 9). For all tasks, the tremor PSD is the highest in pronation-supination, except for the Drawing-Spiral task because the forearm stabilises the movement on the table. Even though the state-space of the wrist shows reduced deflection in flexion-extension, ulnar-radial deviation and pronation-supination, the trajectory of the hand in space shows an improvement of the control over the task execution. However, the reduction in the range of motion in pronation-supination is an unexpected but beneficial side effect of the orthosis. We hypothesise that this reduction is introduced by geometrically restricting the ulna and radius by the inflated air-structure, leading to the constraints in pronation-supination. However, the orthosis performance in Pouring and Drinking does not only rely on this effect, since the PSD reduction of the wrist relative to the forearm (combined movement of flexion-extension and ulnar-radial deviation) is significant, 87 and 81%, respectively.

The calculated Power Spectral Density is an estimation made according to the Welch–Bartlett method, which may vary from the actual tremor power and is influenced by recording duration, window selection and degree of smoothing [45]. Execution duration and, therefore, recording duration can fluctuate by inter and intra-subject variation. Measurement errors, such as skin and friction artefacts, can further influence accuracy. The training effect caused by the same sequence of tests was considered as a low bias because the performed tasks of daily living are assumed to already be well-trained. Further influences on the results could be due to psychological (stress) or physiological (fatigue at the end of the session) effects from the patient. The placebo effect may even occur since we could not apply a control condition. It can, therefore, be hypothesised that the suppression performance for each task will be different for each wearer.

It was measured that voluntary movement peaks (0–2 Hz) are significantly suppressed for the Drinking and Drawing-Spiral tasks by 68% (±35%) and 48% (±27%), respectively, whereas no significance was found for the other tasks. Considering the low power level of the voluntary movement when compared to the tremor, the absolute suppressed voluntary movement power was lower than the tremor (with 72.1 deg^2^/s^3^/Hz for voluntary movements and 1243.4 deg^2^/s^3^/Hz for tremors in the Drinking task). Movements of the wrist have been shown to be compensated by the shoulder in wrist orthoses, making up for the suppressed voluntary movements [53, 54]. In this context, an overload of the shoulder could be a potential risk for the use of such devices in the long-term. The analysis shows the orthosis performance in the selected ADL for which it was designed. However, rest tremor was not evaluated within this study and could be a subject for future research. We hypothesise that the performance of the orthosis for rest tremor will be similar to its performance in this study investigating action tremor in ADL. For the orthosis rest tremor performance, the drawback of suppressing voluntary movements would drop out.

In comparison to the hand cuff by Kalaiarasi et al., which uses a semi-active pneumatic system to suppress tremor and achieves a tremor reduction of 31% of the acceleration amplitude [27], our system achieves a tremor peak reduction from 74 to 82%, analysed with PSD for the three significant tasks of Drinking, Pouring and Drawing-Spiral. Taking the average efficacy of all six tasks (considering 0% as being non-significant), we reduce tremor by 39%. However, it is unclear with what kind of tasks or movements Kalaiarasi et al. evaluated their orthosis. The tremor suppression performance was confirmed by the subjective tremor-reducing perception by the patient. Unfortunately, Kalaiarasi et al. did not provide further performance analysis for further comparisons. To the best of our knowledge, we are the first to publish differentiation of tremor suppression efficacy for different ADL. A systematic literature review by Fromme et al. showed that the cross method average tremor suppression efficacy is 63%, which indicates that our soft, textile, integrated orthosis reduces tremor for the tasks of Drinking, Pouring and Drawing-Spiral in a similar magnitude to existing, conventional systems, but with higher performance at low weights and, thus, higher comfort [19].

### Wearability

#### Physical comfort

With the textile-based TAPO, physical comfort is ensured by thermophysiological comfort, sensory comfort and ergonomic comfort. Ergonomic comfort was achieved by manufacturing the glove in different sizes, leading to a better fit to an individual user’s shape. The integrated materials of the gloves are all compliant and, therefore, the device does not restrict the user’s voluntary movements, degrees of freedom or natural workspace when deflated, while being lightweight at 33 g. The mechanism avoids friction and shear load, whereas normal loads were minimised by maximising the size of the air-structure and distributing the forces over a larger skin surface. The air-structure membranes RET value of 11 m^2^Pa/W is lower than the referred 21 m^2^Pa/W for medical applications, indicating high thermophysiological comfort performance.

#### Wearability

Three human-centred design requirements have been taken into account for the design: usability, reliability and aesthetics. An additional wearability design criterion is the performance efficacy. The TAPO tremor reduction efficacy for the significant tasks reaches 74 to 82%, whereas for a combined reduction efficacy it reaches 39% (non-significant corresponding to 0%). These efficacies are in the same magnitude as the systems described in the literature. The usability of the device is very simple: after an intuitive don, similar to a regular glove, the wearer can either inflate or deflate the orthosis to the preferred inflation state for a chosen situation. The simplified don and doff is important for elderly people with increasing finger joint stiffness [55] and mobility restrictions caused, for example, by osteoarthritis which shows increased prevalence with aging [56]. The inflation could either be done by hand ball pump or a small, lightweight electrical pump with a switch. The time taken to fully inflate TAPO from deflated state with a hand ball takes up to 8 pumps (approximately 10 ml per pump), whereas with an electric pump used for blood pressure metering (e.g. SP V 6TC27M RO-D 6 Vdc, Schwarzer Precision, Essen, Germany, with the dimension ⌀27 mm × 58 mm and weight of 62 g) it would take up to 5 s. However, it is a time consuming process and the device’s frequency of use will depend on the user’s perception: how effective is it for him and is the inflating process worth it, from the point of view of the anticipated daily activity? If the orthosis shows no major safety harm for the user and has significant tremor suppression efficacy for certain tasks, then this is indicative of a reliable orthosis. Pressure points caused by tightening the TAPO velcro too tightly is the only identified potential source of hazard for the wearer. We have designed an unobtrusive tremor suppression orthosis by using textiles and a membrane-based air-structure, which can, in principle, be added to ordinary commercial gloves. Therefore, the visual appearance of the device and properties of the inflation mechanism can be adapted and customised to the individual user’s preferences and needs in future.

## Conclusion

We have developed a manual, task-adjustable, textile-based, passive orthosis (TAPO), which suppresses involuntary, tremorous movements using an integrated air-structure and a new laser welding method. The user is able to specifically activate and adjust the orthosis, by inflating it, for certain tasks and ADL which, to the best of our knowledge, no currently commercially available medical orthosis is capable of doing. A proof-of-concept case study showed that the orthosis reduced tremor power significantly for three out of six tasks (Drinking, Pouring and Drawing-Spiral) with a tremor suppression efficacy of 74 to 82% for these three tasks. By digital analysis of the drawn spirals, significant tremor reduction for the task Drawing-Spiral was confirmed, showing a potential for fine motor control movements. One of the key advantages of this new orthosis is that each individual user can decide whether to inflate the air-structure for each task and adjust the stiffness to his needs and preferences.

In further trials, the efficacy of the device will have to be checked for other patients. It may be expected that this tremor suppression efficacy may vary with the amplitude and the frequency of the tremor. One of the big advantages of this new orthosis is that each user can choose for which task the orthosis will be enabled and then individually adjust the air pressure, taking an individual’s comfort needs into account and changing restrictions in the voluntary movements, while maximising the tremor reduction. Thus, each wearer may choose an optimal balance between comfort and performance of the orthosis.

With our concept, we were able to provide a case study that showed that a soft, passive orthosis for the wrist (which is considered as the most complex and poorly understood joint of the body [57]) can have improved comfort and wearability, compared to previous rigid and bulky research prototypes, while providing tremor suppression in the same magnitude. This air-structure technology could also be applied in different degrees of freedom, like fingers, and for other movement disorders, like spasticity, or even be integrated into future exoskeletons and exosuits for the implementation of variable stiffness in the system. In future, the concept of textile integrated air-structures can also be used to stiffen human joints and also stiffen human-robot-interaction (in the same way as cuffs) because of its potential to enhance the convenience and comfort for the wearable robot [58].

## Data Availability

The datasets generated are available from the corresponding author on reasonable request.

## Abbreviations

ADL: Activities of daily living
ET: Essential tremor
PD: Parkinson’s disease
PSD: Power spectral density of tremor (rad^2^/s^3^/Hz)
RET: Resistance to Evaporating Heat Transfer
TAPO: Task-adjustable passive orthosis
